# Development and Experimental Assessment of Friction-Type Shear Connectors for FRP Bridge Girders with Composite Concrete Decks

**DOI:** 10.3390/ma15093014

**Published:** 2022-04-21

**Authors:** William G. Davids, Dante Guzzi, Andrew P. Schanck

**Affiliations:** 1 Department of Civil and Environmental Engineering, University of Maine, 5711 Boardman Hall, Orono, ME 04469, USA; 2 HNTB, 82 Running Hill Road, South Portland, ME 04106, USA; dguzzi@comcast.net; 3 Advanced Structures and Composites Center, University of Maine, 35 Flagstaff Road, Orono, ME 04469, USA; andrew.schanck@maine.edu

**Keywords:** FRP composites, shear connectors, fatigue testing, composite beams

## Abstract

This paper details the development and experimental assessment of a friction-type connector, designed to transfer shear flow between the top flange of a fiber-reinforced polymer (FRP) tub girder and a composite concrete deck for bridge applications. In contrast with previously used bearing-type connectors, this system relies on a deformed FRP surface to transfer shear via direct interlock with the concrete deck. The connector is materially efficient, simple to fabricate, can be used with lower-grade structural or stainless-steel fasteners, and provides a high degree of interface stiffness. Six compression-shear specimens were tested to assess the connector fatigue resistance and ultimate connection strength. Additionally, two short beam specimens were tested in three-point bending, one of which was subjected to fatigue loading. Based on the compression-shear tests and short beam tests, the connection exhibited strength exceeding that predicted by AASHTO for frictional concrete-concrete connections. The connection strengths were significantly greater than the factored demand required by AASHTO for a typical model FRP bridge girder. The cyclic loading of the connection in both compression-shear and beam bending showed that connection stiffness and strength do not significantly degrade, due to the application of 1 × 10^6^ to 6 × 10^6^ cycles of traffic-induced factored fatigue load.

## 1. Introduction

The low weight and high durability of fiber-reinforced polymer composites (FRPs) make them attractive for infrastructure applications, such as bridges. For these reasons, the use of FRP concrete reinforcing in corrosive environments continues to increase and its design and specification are well understood and codified [1,2]. Similarly, design codes exist for the strengthening of beams with externally bonded FRP [3,4], and FRP wraps are regularly used to increase column strength and ductility [5,6,7,8].

Other uses of FRP in bridge superstructure members are relatively rare. One notable example is concrete-filled FRP arches for short-span buried bridges [9,10], which have been used in the construction of more than 20 structures to-date [11], and for which an AASHTO guide specification exists [12]. However, buried structures are suitable for a relatively small percentage of sites due to their geometry, with slab-on-girder bridges being much more common. Despite this, relatively few FRP vehicular girder bridges have been developed and deployed to-date. A vacuum-infused FRP girder with a trapezoidal section made composite with a concrete deck, by bonding pultruded FRP I-beams to the girder top flange, was reported by Gutierrez et al. [13] and installed in a bridge in Spain [14]. Ziehl et al. [15] developed an FRP tub girder used for a demonstration bridge in Texas, USA that was partially filled with foam and contained transverse stainless steel bars, attached through holes in the webs to provide composite action with the concrete deck. Siwoski et al. [16] and Siwoski and Rajchel [17] documented the development and implementation in Poland of a vacuum-infused, glass-carbon bridge tub girder that relied on steel stud connectors between the girder and deck that were secondarily bonded to the girder top flanges. Most recently, Davids et al. [18,19] reported the design and development of a 22.9 m span, FRP tub girder bridge that was constructed in Maine, USA in late 2020 that relied on bearing-type, bolted stud connections between the girder flange and concrete deck.

One important common element in these four FRP girder bridges is their reliance on composite action between the girders and concrete deck. This composite action—achieved by the transfer of shear flow and minimization of slip between the girder top flanges and concrete deck—greatly increases material efficiency, through a reduction in the bending stresses in the FRP, and significantly enhances flexural stiffness. For the same reasons, virtually all modern steel and concrete bridge girders behave compositely with a concrete deck. However, each FRP girder bridge described above relies on a distinctly different method of achieving composite action that required novel design and special detailing. This special design and detailing is not necessary with conventional steel and concrete girder bridges, for which codified designs that provide sufficient fatigue and strength resistance are well-developed. The system documented in reference [20] was relatively straightforward compared to the connectors used in other FRP girder bridges, as it relied on conventional steel bolts and did not require any secondary bonding to the FRP or welding. However, because of its direct reliance on the fasteners to transfer shear and the critical nature of the fatigue limit state, high strength ASTM A490 bolts were required. Furthermore, the connection required a large number of bolts, and holes in the flanges had to be accurately machined to ensure a tight fit between the bolts and the FRP flanges, to promote bearing and minimize slip. Finally, an extensive experimental assessment of the connection strength and fatigue resistance was required, as reported in reference [20], to ensure the connector met the design requirements.

**Figure 1:**
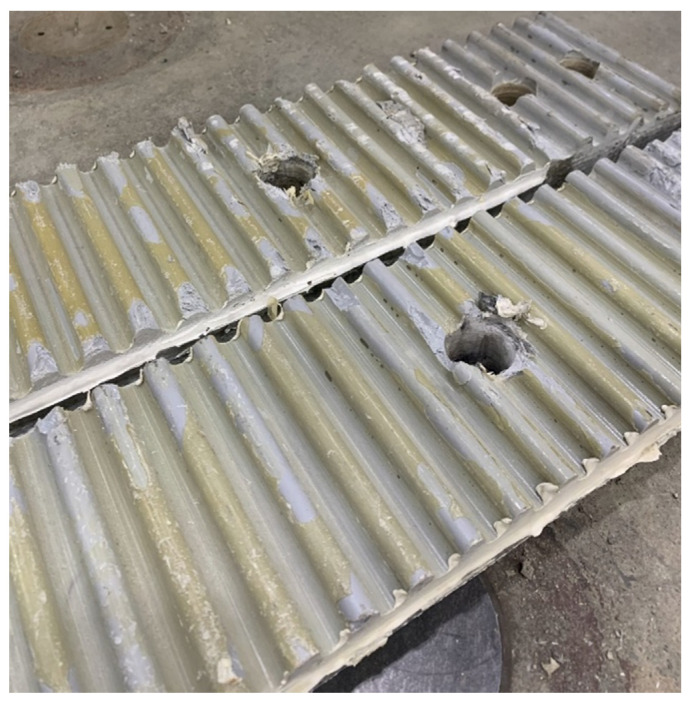
Deformed FRP Girder Top Flanges Used in Shear-Friction Connection.

This study reports the development of a novel, more efficient, friction-type FRP girder-deck shear connector that relies on surface deformations and mechanical interlock between the girder and deck to transfer shear flow, instead of direct fastener bearing. This connection, as seen in Figure 1, is simple to manufacture, permits the use of a lower grade or stainless steel fastener, and requires significantly fewer fasteners than a bearing-type connection. The originality and significance of this research derives from the carefully designed experimental program and the usefulness of this connector in an entirely new type of bridge girder. Following a brief description of the FRP girder used to develop connector demands, the new shear connector is presented, and laboratory compression-shear tests of four specimens that quantify the traffic-induced fatigue and strength characteristics of connectors employing low-grade structural bolts are detailed. To further validate the connector performance, two short-span beams, designed to fail in shear at the girder-deck interface, were tested to failure in the laboratory, with one specimen subjected to prior fatigue loading. Finally, a version of the connector using a stainless steel threaded rod is presented and experimentally assessed with strength and fatigue testing of two additional specimens. The results of the experimental program clearly demonstrate the adequacy of the connector system, with either the conventional structural steel bolts or stainless steel threaded rod, and this connection is slated for use in multiple FRP-girder bridges to be constructed in 2022 and beyond.

## 2. Description of Model FRP Girder

**Figure 2:**
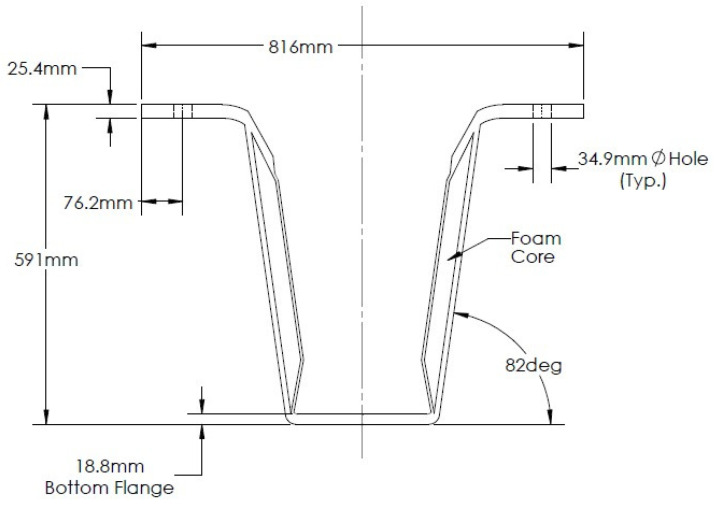
FRP Test Girder Cross-Section.

The FRP girder section shown in Figure 2 was used as the basis for the initial connector development and testing detailed in this paper. The important characteristics and properties of the section, along with results of its analysis, are reported here to support the development of the connector testing program. The girder was proportioned to support Strength I loading per current AASHTO *LRFD Bridge Design Specifications* [21], assuming a 12.5 m simple span model structure having a 9.14 m overall width and 4 girders spaced at 2.29 m on the center. The girder’s concrete deck consisted of precast panels that were grouted to the shear connectors, using a self-consolidating concrete mix with a 9 mm maximum aggregate size. The design loading consisted of self-weight plus 0.54 lanes of AASHTO HL-93 live load with impact per girder, and 0.51 lanes of AASHTO traffic-induced fatigue loading, including impact per girder. All Strength I HL-93 truck loads were increased by 25%, per Maine Department of Transportation requirements. A 75 mm asphalt wearing surface was assumed to be applied as a minimum superimposed dead load.

**Figure 3:**
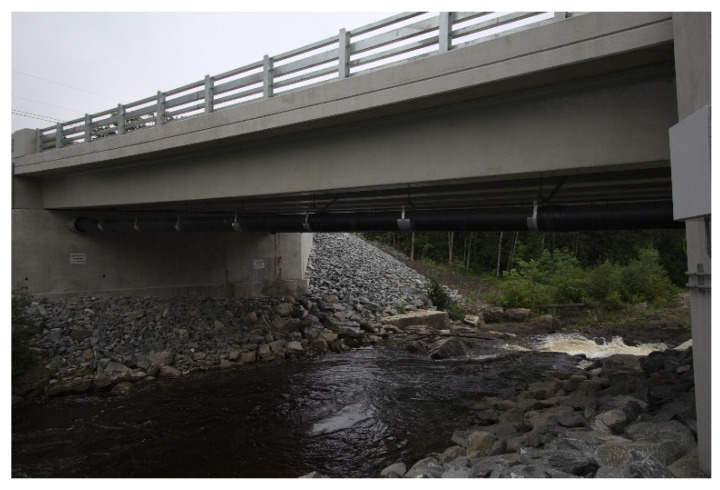
22.9 m Span FRP Girder Bridge in Hampden, Maine, USA Constructed in 2020.

The member was fabricated by vacuum infusion using a 3D-printed polymer female mold, and had solid E-glass top flanges, consisting of alternating layers of +/−45° and 0° E-glass, foam-core webs with balanced +/−45° E-glass face sheets, and a bottom flange, containing uniaxial carbon fabric. Derakane 610c resin produced by Ashland Global of Wilmington, DE, USA—an epoxy-vinylester blend exhibiting excellent durability and toughness that has been successfully used to fabricate hybrid, carbon/glass, concrete-filled FRP tubes used in bridge construction [9,22]—was used for the infusion. The key mechanical properties of the section derived from laminate analysis are summarized in Table 1. It is important to note that the test girder employed first-generation bearing-type shear connectors that were closely spaced at 15.2 cm on the center in each flange. These connectors provided good composite action in the bending test, and the test to failure showed the girder to have a live load bending strength of approximately six times the service live load with impact [18]. However, these connectors exhibited poor fatigue performance in subsequent testing [20]. As a result, the 22.9 m vehicular bridge constructed on the US Route 1A in Hampden, Maine, USA in 2020 (see Figure 3) utilized a second-generation bearing-type connector demonstrated to have sufficient fatigue resistance [20]. An analysis of data from a field live load test of this bridge verified that full composite action was achieved using the second-generation connector [19], although it was reliant on closely spaced high-strength A490 bolts that carried shear transferred through direct bearing on the girder flanges [18,20].

## 3. Friction-Type Shear Connector and Connector Shear Test Protocol

### 3.1. Rationale for and Development of Shear Connector

In contrast with the previously used bearing-type connector, the friction-type shear connector developed here relies on the deformed upper surfaces of the girder flanges, to transfer shear through the mechanical interlock of the concrete deck and flange. The concrete deck (or grout in the case of a precast deck) is cast against the top flange, molding to the flange deformations. This connection is analogous to a shear-friction connection permitted for all concrete construction by both ACI [23] and AASHTO [21]. The design shear strength of an intentionally roughened concrete construction joint, $\emptyset V_{ni}$, prescribed by AASHTO load and resistance factor design (LRFD) [21] is given in Equation (1).

(1) $$\emptyset V_{ni}=\emptyset \left(cA_{cv}+\mu \left(A_{vf}f_{y}+P_{c}\right)\right)$$

In Equation (1), the term $cA_{cv}$ captures cohesion, $A_{vf}$ is the area of reinforcement perpendicular to the shear plane, $f_{y}$ is the yield stress of the reinforcing crossing the plane (limited to 414 MPa), $P_{c}$ is the net compressive force acting on the shear plane, and μ=1 for a purposely deformed surface. Here, as in most design situations, c and $P_{c}$ are taken as zero and the strength reduction factor is ∅= 0.9, so Equation (1) simplifies to Equation (2).

(2) $$\emptyset V_{ni}=0.9A_{vf}f_{y},$$

Assuming a lower-grade, 25.4 mm diameter A325 bolt with a yield strength of 624 MPa, the 414 MPa yield limit controls, and Equation (2) predicts a design shear strength of 189 kN per bolt. The factored AASHTO LRFD Strength I shear flow at the deck-flange interface for the model FRP girder, detailed in the previous section, is 0.404 kN/mm [20], which implies that a connector spacing, s=47 cm, is theoretically sufficient for strength, with an appropriately deformed surface and 25.4 mm A325 bolts used as connectors. This value of s is more than three times larger than the spacing of the bearing-type connectors used in the model FRP girder test. Furthermore, these bolts will not require accurately machined holes in the girder flanges, since the shear transfer will not be through bolt bearing. These observations point to significant potential for a more easily constructed, efficient shear connection.

**Figure 4:**
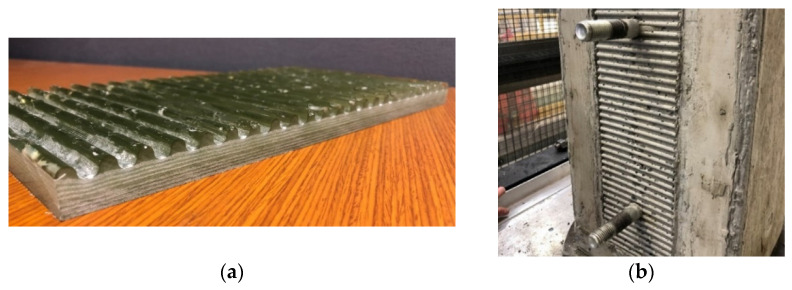
Elements of Ridged Shear-Friction Connection: (**a**) FRP Plate Infused with Ridges Using Caul Plate; (**b**) Concrete Surface Cast Against Ridged FRP Plate.

A remaining challenge is the creation of the deformed surface on the girder top flange, for which at least three methods exist. The deformations can be formed by machining grooves in the flange after girder infusion, by bonding a deformed plate or other protrusions to the smooth flange top surface after infusion, or creating the deformations during the primary infusion. The third option was selected, since it is the least labor-intensive, uses less material, and does not rely on secondary bonding, which creates an additional failure path that might be subject to environmental or fatigue degradation. The deformations were formed using a high-density polyethylene (HDPE) caul plate, with sinusoidal ridges machined across its width on one surface. The minimum amplitude of the sinusoidal ridges required by ACI [23] for intentionally roughened concrete surfaces in shear-friction connections is 6.3 mm. Before the infusion, the HDPE caul plates were placed above the top flange fabric and under the vacuum infusion bag, with the ridges facing downward. During the infusion process, resin flows between the ridges on the caul plate and produces mirror-image sinusoidal corrugations in excess resin, on the upper surface of the girder top flange. To illustrate, Figure 4a shows a single vacuum-infused FRP plate with the sinusoidal ridges, and Figure 4b shows a cured concrete surface that was cast against a ridged FRP plate. It is important to note that the ridged HDPE caul plates do not bond to the vinylester resin when treated with a bond-release agent, and so can be reused for multiple infusions.

### 3.2. Connector Shear Test Protocol

While Equation (2) indicates that the shear connection should have sufficient strength with a large connector spacing and lower-grade bolts, Equation (2) was developed for concrete-concrete connections with reinforcing bars intersecting the shear plane, and the novelty of the ridged FRP shear connection dictates that its capacity should be experimentally verified. More importantly, however, Equation (2) gives no insight into the performance of this connection under the action of traffic-induced fatigue loading. Therefore, two compression-shear specimens were loaded monotonically to failure to assess the connection ultimate strength, and two compression-shear specimens were subjected to cyclic fatigue loading, prior to being monotonically loaded to failure, to assess fatigue-induced damage and post-fatigue strength. This compression-shear test method creates shear flow in the plane of the connection that is easily quantified using equilibrium and, therefore, can reliably and accurately produce the shear flows produced by vehicular loads, at the girder-deck interface of a bridge. This test has been shown by previous studies to accurately assess the performance of other shear connectors, such as perforated steel plates [24,25], headed studs [26], and adhesive bonding [27]. As detailed subsequently, the load protocol defined in this study was designed to precisely mimic the AASHTO factored load effect for traffic-induced fatigue in a typical FRP girder bridge, designed for infinite fatigue life.

**Figure 5:**
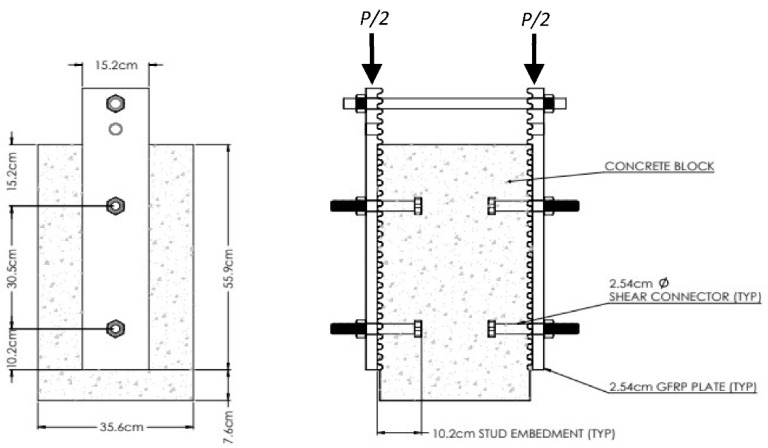
Shear Block Test Specimen Details and Geometry.

The dimensions of the compression-shear test specimens and the test configurations are shown in Figure 5. The concrete block was cast against two opposing ridged FRP plates, with a fiber architecture and dimensions matching the girder flanges. The same self-consolidating concrete, with a 9 mm maximum aggregate size that was used for the previously detailed test girder with precast deck panels and tests of bearing-type connections [18,20], was used. The initial tests of all four specimens used A325 bolts spaced at 30.5 cm, less than the 47 cm theoretically needed for strength, but twice the spacing of the bearing connectors used in the original girder test. (Later in this paper, the use of a stainless steel threaded rod in lieu of A325 bolts is assessed with additional tests.) The FRP plates were coated with form oil, prior to the concrete casting, to eliminate the chemical bond between the ridged FRP and concrete surfaces, and the plates were removed after the block was cured, prior to reinstalling for each test, to verify there was no chemical bond. The fasteners were torqued to 305 N-m and independent tests of instrumented bolts by Guzzi [20] demonstrated to produce a bolt pre-tension of 227 kN, the value required by AASHTO [21] for a slip-critical connection. To produce identical shears of P⁄2 on each side of the connection as shown in Figure 5, the specimens were loaded by a hydraulic actuator reacting on the center of a single steel plate, supported by the protruding portions of both FRP connection plates. The instrumentation consisted of a calibrated load cell, as well as four linear variable differential transformers (LVDTs) that measured relative slip during the fatigue test, one at each fastener. For the strength tests, PONTOS digital image correlation was used to measure the relative slip between the FRP plate and the concrete block near each bolt. These and all the tests detailed later in this paper were conducted indoors, at a temperature of approximately 20 degrees C. The humidity was not controlled, and did vary seasonally and, to a lesser extent, daily, depending on the time of year. However, the material properties of the concrete and FRP are not expected to vary significantly with normal changes in temperatures and humidity experienced by a typical bridge.

The uniaxial, compressive fatigue loading regime was based on AASHTO’s requirements for infinite fatigue life (Fatigue I limit state) for welded shear studs, used in composite steel girder bridges. While welded studs can be expected to have a different fatigue life than the connection developed here, applying welded stud provisions should be conservative, since welded studs will be more prone to fatigue failure, due to the presence of residual stresses caused by welding. The AASHTO infinite fatigue life provisions were derived from reference [28], which gives Equation (3) for $Z_{r}$, the fatigue strength of a steel shear stud of diameter *d* corresponding to an infinite fatigue life.

(3) $$Z_{r}=7.0\frac{\pi }{4}d^{2}$$

For finite fatigue life, AASHTO [21] specifies the calculation of stud capacity, $Z_{r}$, corresponding to the *N* fatigue cycles using Equation (4).

(4) $$Z_{r}=\left(34.5-4.28\log N\right)d^{2}$$

Substituting $Z_{r}$ from Equation (3) into Equation (4) gives $N\approx \;6\times 10^{6}$, and therefore, 6 × 10^6^ cycles of factored AASHTO LRFD Fatigue I loading were applied to the cyclically loaded specimens at a rate of 4 Hz. A single fatigue test required nearly 18 days to complete.

The fatigue load range, $P_{LR}$, applied to the test specimens was determined using the AASHTO LRFD [21] provisions for the Fatigue I load case, which applies for details intended to have an infinite fatigue life and incorporates a fatigue live load factor of 1.75 and an impact factor of 15%. The applicable live loading is 51% of an HL-93 truck for the previously detailed model bridge girder [18]. We note here that a real bridge will typically be subjected to a larger number of lower-load, traffic-induced fatigue cycles, corresponding to service load truck traffic, during its design life. However, AASHTO design guidelines are based on the application of 1.75 times the service load for a smaller number of cycles, $N\approx \;6\times 10^{6}$, to produce an equivalent amount of fatigue damage. The Fatigue I shear flow, $f_{v}$ = 0.15 kN/mm, at the girder-deck interface was calculated with Equation (5), where *V* is the factored fatigue shear force, $Q=7.57\times 10^{3}\;{\mathrm{cm}}^{3}$ is the first moment of the un-cracked deck area about the girder neutral axis, and $I=4.64\times 10^{5}$ cm^4^ is the moment of inertia of the transformed girder section, expressed in terms of concrete. The assumption of the linearly elastic response of the full cross-section inherent in Equation (5) is valid, given that the bending moments are negligibly small near the supports where the shear flow is largest. Equation (6) was used to compute the cyclic load range, $P_{LR}$ = 167.6 kN, applied to the compression shear specimen, where *l* is the 55.9 cm length of each ridged FRP plate in contact with the concrete block.

(5) $$f_{v}=\frac{VQ}{I}$$

(6) $$P_{LR}=2f_{v}l,$$

The minimum load applied to the specimen $P_{min}$ was fixed at 44.5 kN, giving a maximum load of $P_{max}=P_{min}+P_{LR}=$ 212 kN. The minimum load, $P_{min}$, represents the effect of permanent, superimposed dead loads, and 44.5 kN is approximately 70% larger than the superimposed dead load, due to the assumed 75 mm asphalt wearing surface and fraction of a typical bridge rail. The additional permanent load builds in some conservatism, to account for the possibility of additional loads on in-service structures, such as utilities, thicker wearing surfaces and heavier railings. Table 2 summarizes details and loads applied to the four compression-shear connection test specimens employing A325 fasteners, as well as the concrete compressive strength, $f_{c}^{\prime }$, on the day of strength testing taken from standard cylinder tests. Specimen S4 was cast from a different batch of nominally identical concrete that had a significantly lower concrete strength.

## 4. Connector Test Results

**Figure 6:**
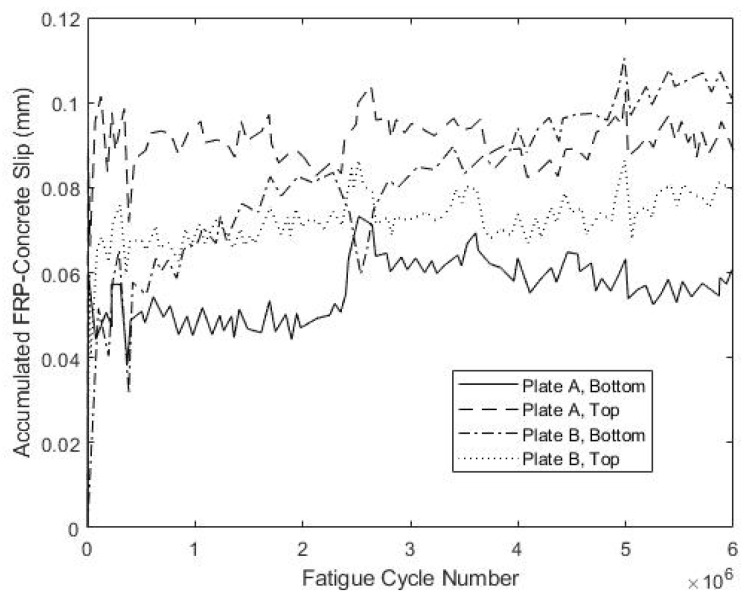
Fatigue-Induced Slip at FRP-Concrete Interface for S1.

Both specimens S1 and S2 withstood the full 6 × 10^6^ cycles of fatigue loading, while exhibiting little stiffness degradation or apparent damage. To illustrate, Figure 6 shows the total measured slip near each fastener, corresponding the maximum load of 212 kN measured over the course of 6 × 10^6^ load cycles for specimen S1. These data indicate that the maximum slips were very small, on the order of 0.1 mm, and tended to stabilize near their peak value early in the fatigue regime. This is consistent with very little damage accumulation and good shear transfer between the concrete and FRP. The slips observed for specimen S2 had greater variability at different fasteners, but were similarly small, ranging from 0.02 to 0.13 mm, and also stabilized early in the fatigue regime.

**Figure 7:**
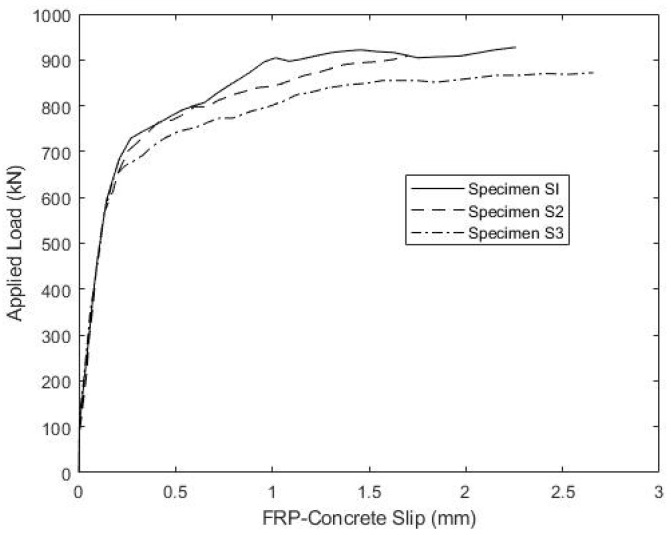
Shear Connection Load-Slip Response under Monotonic Loading to Failure.

**Figure 8:**
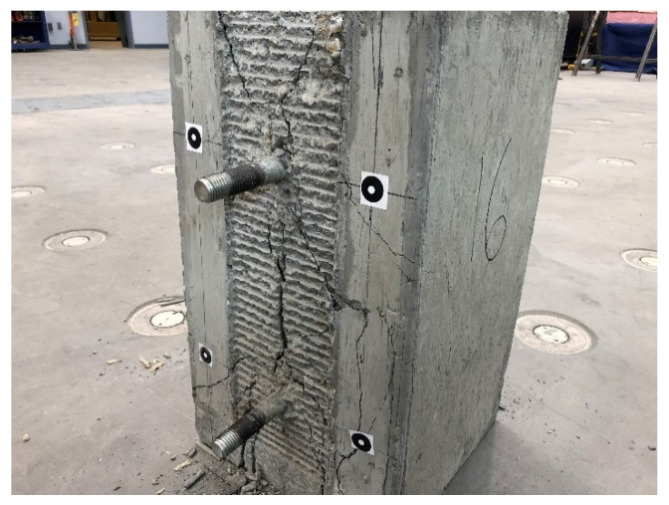
Compression-Shear Specimen after Loading to Failure.

Following fatigue testing, all four compression-shear specimens were loaded monotonically to failure. The tests were conducted in displacement control at a constant displacement rate, producing failure in approximately two minutes. Figure 7 shows the results for both S1 and S2 that were initially subjected to fatigue and the non-fatigued control specimen S3. Unfortunately, slip data were lost for control specimen S4 due to an equipment malfunction, although the maximum load was recorded. The results indicate very similar responses for all three specimens, which confirms the previous observation that fatigue caused little damage to S1 and S2. The response of all three connections is nearly identical, up until about 600 kN of load, where their response diverges. The failure mode for all four specimens was the fracture of the concrete ridges, while the ridges in the FRP connection plates remained intact. This was true for all specimens, suggesting both that the FRP ridges were able to withstand the expected ultimate shear flows and that the connection design was controlled by concrete shear strength. To illustrate, Figure 8 shows the failed concrete ridges on a compression-shear specimen post-failure, following the removal of the FRP plates. The bolts were not bent or otherwise deformed, and although there is a vertical crack in the concrete block, there was no evidence of a bearing failure of the concrete or the FRP plate near the bolts.

The failure loads for all four connectors are given in Table 2. The failure loads for specimens S1–S3 fall within a tight range of 872 to 927 kN, while specimen S4 failed at a significantly lower load of 698 kN. This is consistent with the observed failure mode of the concrete ridge shear, as the concrete compressive strength of specimen S4 was 40% less than that for specimens S1–S3. Another important observation is that the strengths of the fatigued specimens S1 and S2 were slightly more than the strength of the control specimen S3. This indicates that fatigue cycling did not reduce connection capacity, and agrees with the previous observations of very small slip accumulation during fatigue and no loss of connection stiffness due to fatigue.

It is also valuable to compare the measured strength with the AASHTO Strength I demand and the capacity predicted by Equation (2). To facilitate this, Table 2 also reports shear flows at failure, which are the maximum applied loads divided by the length of the plates in contact with the concrete, 2l= 1118 mm. These shear flows at failure range from 0.624 to 0.829 kN/mm, which are 54% to 105% greater than the 0.404 kN/mm AASHTO LRFD Strength I factored loading for the model bridge, respectively. For the 30.5 cm test specimen connector spacing, Equation (2) predicts a capacity of 0.619 kN/mm. This value is less than the shear flows at failure for all specimens, although the observed strength of specimen S4 exceeds this by only 0.8%. This is likely because the concrete compressive strength,$f_{c}^{\prime }$, of specimen S4 was significantly lower than that of S1–S3. However, both ACI [23] and AASHTO [21] consider concrete shear strength to be proportional to $\sqrt{f_{c}^{\prime }}$, indicating that the shear flow at failure, $f_{v}^{u}$, normalized by $\sqrt{f_{c}^{\prime }}$, provides a rational basis for comparison across specimens with different concrete strengths. The quantity $f_{v}^{u}/\sqrt{f_{c}^{\prime }}$ is given in the last column of Table 2, and shows a tight grouping of this normalized failure quantity of between 85 and 90 for all specimens. This is consistent with the observed shear failures of the concrete ridges, although the small number of specimens and the fact that only two concrete strengths were assessed prevent drawing any firm conclusions.

It is also valuable to assess connection stiffness, as it helps to better understand the degree of composite action the connection can provide. For the linear range of load-slip data up to ~600 kN, as shown in Figure 7, the average stiffness of specimens S1–S3 is 4250 kN/mm. In contrast, similar tests of bearing-type connectors with four, A490 fasteners exhibited an average stiffness of 625 kN/mm [18], which is 85% less than the 4250 kN/mm observed here. The greater stiffness of the ridged friction-type connection will result in a higher degree of composite action in an FRP beam with a concrete deck.

Taken as a whole, the shear connector tests indicate good performance, with adequate strength and high stiffness. However, connector capacity is dependent on the strength of the concrete ridges. The impact of the ridge size is unknown, although intuitively, larger ridges might provide better performance. Furthermore, since the A325 bolts experience no bearing and their yield stress exceeds the upper limits specified by AASHTO, it might be feasible to use lower strength or even stainless steel studs, with threads in the shear plane. The performance of this connection with stainless steel studs used in conjunction with larger ridges is experimentally assessed later in this paper.

## 5. Short Beam Test Specimens and Test Protocol

While the compression-shear tests reported in the last section provide important information on connector strength and stiffness, it is also useful to assess the connector performance in a beam. To this end, two short FRP beams were fabricated with ridged top flanges, concrete decks, and the same A325 bolts spaced at 30.5 cm as used in the connection tests. The two beams were nominally identical, but specimen B1 was initially fatigue cycled in three-point bending; both beams B1 and B2 were subsequently loaded to failure in three-point bending. The beam cross-section was sized to ensure that the flange-deck shear connection would fail before the beam experienced bending or web shear failure, to assess connection strength as detailed in reference [20].

**Figure 9:**
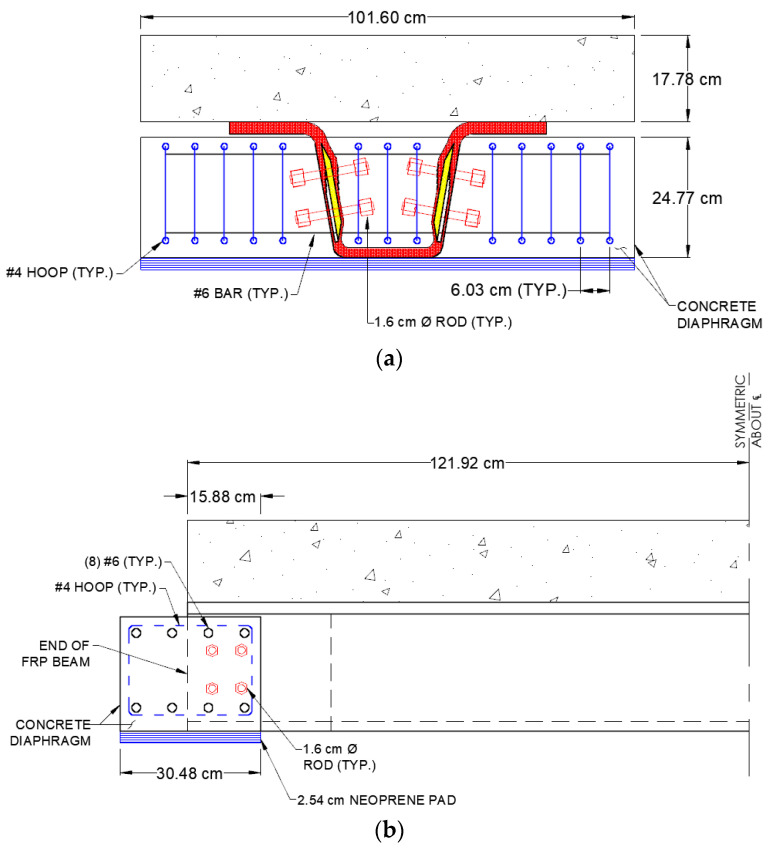
Details of Short Beam Specimens: (**a**) End Elevation; (**b**) Details of Short Beam Specimens.

Table 3 summarizes the dimensions of the beams; the same E-glass and carbon fabric architecture and foam core webs used for the model beam detailed in Section 2 were used here. Figure 9 is a dimensioned drawing of a specimen, showing the 17.8 cm thick concrete deck and details of the beam connection to the end concrete diaphragm. The concrete deck had longitudinal and transverse steel reinforcing, typical of that found in a bridge deck, and was cast from the same self-consolidating mix used for the shear block specimens. The deck concrete compressive strength, $f_{c}^{\prime }$, on the day of beam strength testing was 77.4 MPa, based on standard cylinder tests. The critical computed cross-sectional properties needed to compute shear flows at the deck-girder interface using Equation (5) are the first moment of the concrete deck taken about the neutral axis of the section Q = 4503 cm^3^ and the transformed section moment of inertia I = 162.2 × 10^4^ cm^4^, as reported by Guzzi [20].

**Figure 10:**
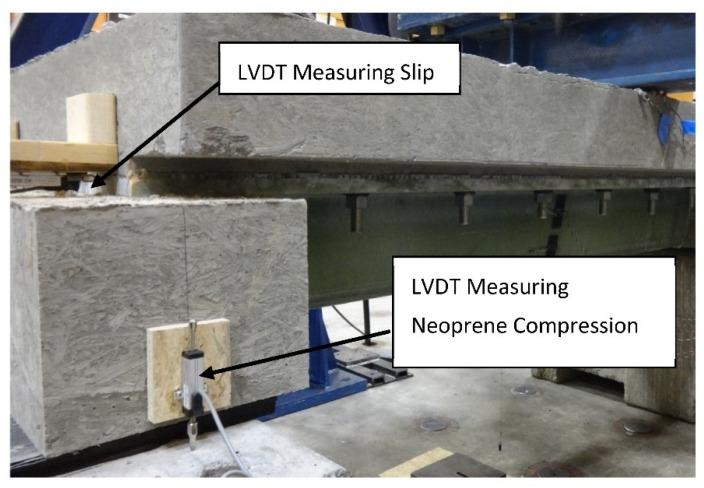
Short Beam Specimen Instrumentation at Support.

Beam B1 was initially subjected to 1 × 10^6^ cycles of load applied at the middle of the 2.44 m span. The three-point bend configuration produced nearly uniform shear flows at the deck-girder interface over each half of the span. As discussed previously, an infinite fatigue life corresponds to 6 × 10^6^ fatigue cycles, but the largest frequency at which loads could be applied without introducing dynamic effects was 1 Hz, precluding the application of more than 1 × 10^6^ fatigue cycles in a reasonable amount of time. Additionally, the compression-shear fatigue tests indicated that no significant damage accrued after a few thousand load cycles, and this behavior could be reasonably assumed to extend to the girders. The loads were cycled sinusoidally from 33.1 kN to 249 kN, which according to Equation (5), produced the same target Fatigue I stress range in each flange of 0.15 kN/mm used in the compression-shear tests. The minimum load of 33.1 kN was derived from the previously defined target superimposed dead load of 0.023 kN/mm. Following fatigue cycling, beam B1 was loaded in displacement control at a constant rate to failure. Beam B2 was a control specimen loaded to failure identically to beam B1, but was not initially fatigued. All load was applied via a 1330 kN hydraulic actuator and load was recorded with a calibrated load cell. As shown in Figure 10, additional instrumentation included two LVDTs at each girder end to measure slip between the concrete deck and girder flanges, and two LVDTs at each support to measure the vertical compression of the neoprene bearing pad. An LVDT was also installed at midspan to measure gross beam vertical displacement.

## 6. Beam Test Results

**Figure 11:**
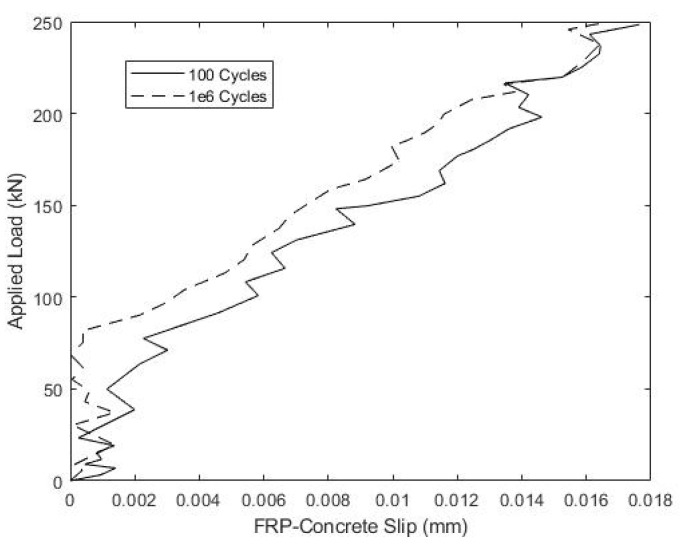
Beam B1 Service Load-Slip Behavior during Fatigue Cycling.

**Figure 12:**
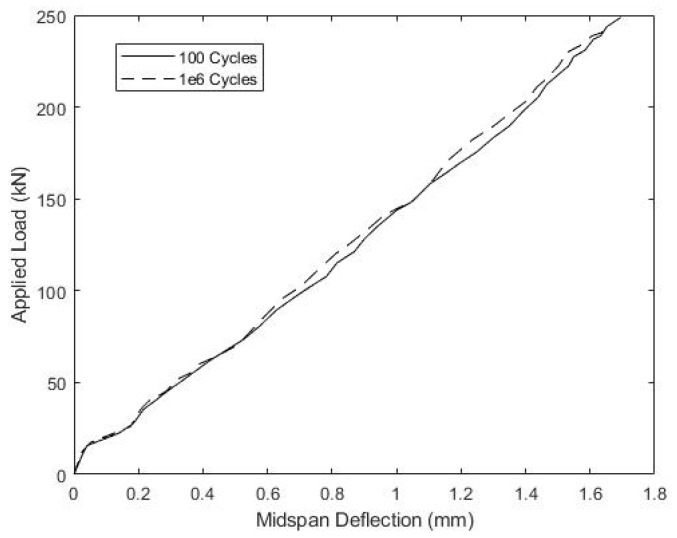
Beam B1 Service Load vs Mid-span Displacement during Load Cycling.

Specimen B1 withstood the full 1 × 10^6^ fatigue cycles, and Figure 11 illustrates the relationship between the applied load and slip up to the full fatigue load of 249.3 kN after 100 and 1 × 10^6^ fatigue cycles. The small recorded slips and similar slip magnitudes during both tests indicate no significant damage or loss of composite action due to load cycling. Consistent with this, Figure 12 shows nearly identical, linear load versus mid-span displacement for beam B1 after 100 and 1 × 10^6^ fatigue cycles.

**Figure 13:**
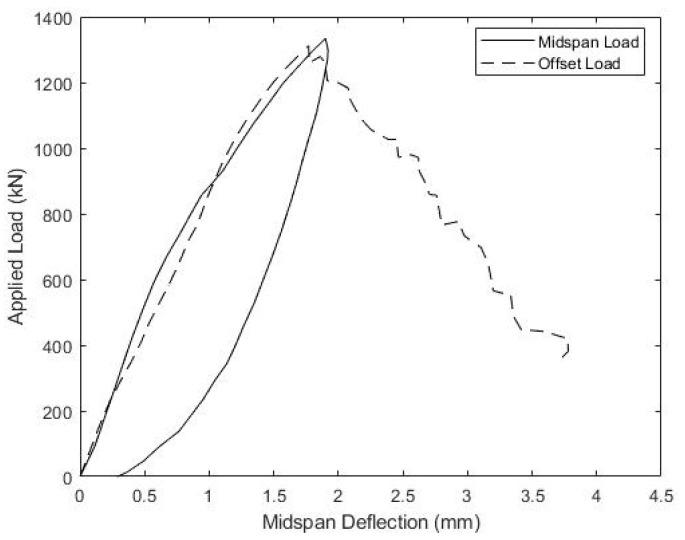
Beam B1 Ultimate Load-Displacement Response.

**Figure 14:**
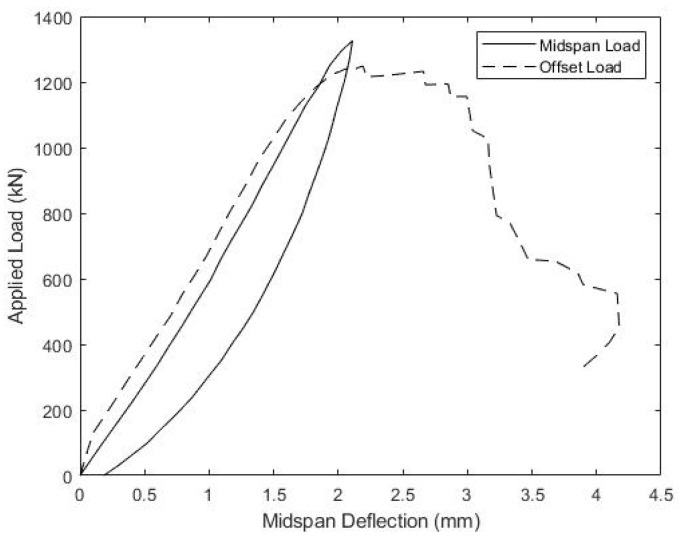
Beam B2 Ultimate Load-Displacement Response.

**Figure 15:**
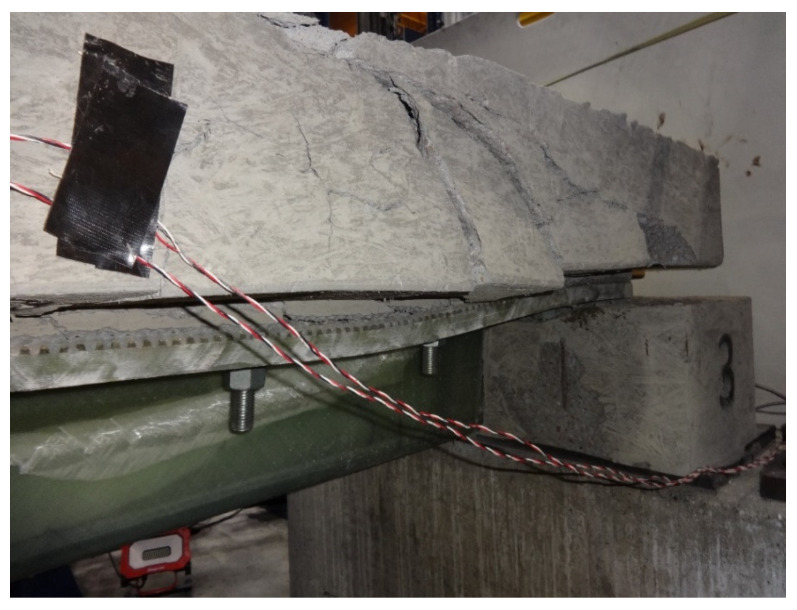
Post-Failure Image of Beam B1.

The load versus mid-span displacement results, gathered during the strength testing of beams B1 and B2, are given in Figure 13 and Figure 14, respectively. Beam B1 was initially loaded with the actuator positioned at mid-span, as represented by the solid curve in Figure 13, but the maximum actuator capacity was reached before catastrophic failure, and the beam was then unloaded. In order to increase internal shear between the load point and one support to induce failure, the actuator was subsequently shifted 30.5 cm closer to one support. This increased the maximum shear in the beam by 25%, from P/2 to 0.625P, for a given actuator load P. The results of the offset load test are given in Figure 13 by the dotted curve, and shifting the actuator was successful at producing failure. For consistency, the same protocol of initially loading at mid-span, followed by offsetting the actuator 30.5 cm closer to one support, was also used to test beam B2, and its load-displacement response is shown in Figure 14. Figure 15 includes a photo of B1 after failure highlighting the diagonal cracking of the deck that coincided with the failure of the concrete ridges.

The load-displacement response of beams B1 and B2 are similar, with both specimens sustaining the full actuator load under mid-span loading, followed by failure under offset loading. Beam B1 carried a peak load of 1315 kN during the offset load test, which corresponded to a maximum shear force of 822 kN, whereas beam B2 carried a peak force of 1248 kN, producing a maximum shear force of 780 kN. These shear forces at failure can be used to estimate the maximum shear flow, $f_{v}^{u}$, at the girder-deck interface with Equation (5), which gives $f_{v}^{u}$ = 0.898 kN/mm for beam B1 and 0.853 kN/mm for beam B1. These values are both higher than the shear flows at failure taken directly from the compression-shear specimens S1–S3 (Table 2), despite specimens S1–S3 having a slightly higher concrete compressive strength. Consistent with this, the normalized shear flows at failure, $f_{v}^{u}/\sqrt{f_{c}^{\prime }}$, are 102 and 97 for B1 and B2, respectively, both higher than observed for compression-shear specimens S1–S4. One possible reason for these higher, directly observed and normalized strengths is the weight of the concrete deck reacting downward on the shear plane in the beam tests, which tends to increase the shear capacity, as shown in Equation (1). This effect was not present in the compression-shear tests where the flange plates were oriented vertically, nor was it accounted for when predicting connection capacity.

**Figure 16:**
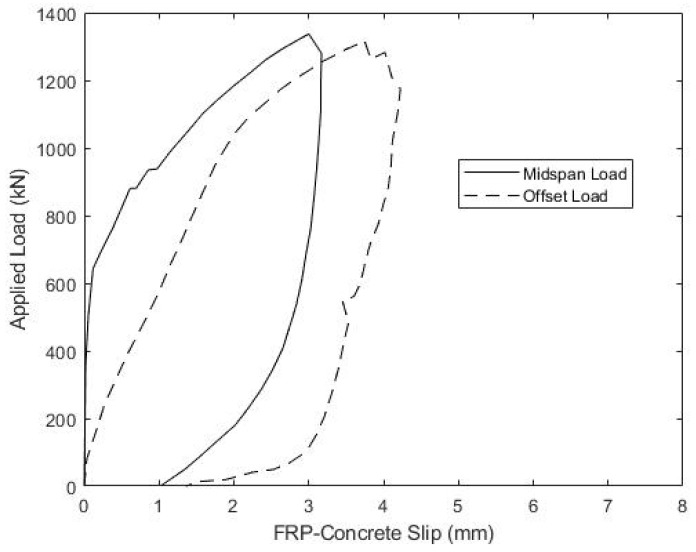
Beam B1 Ultimate Load-Slip Response.

**Figure 17:**
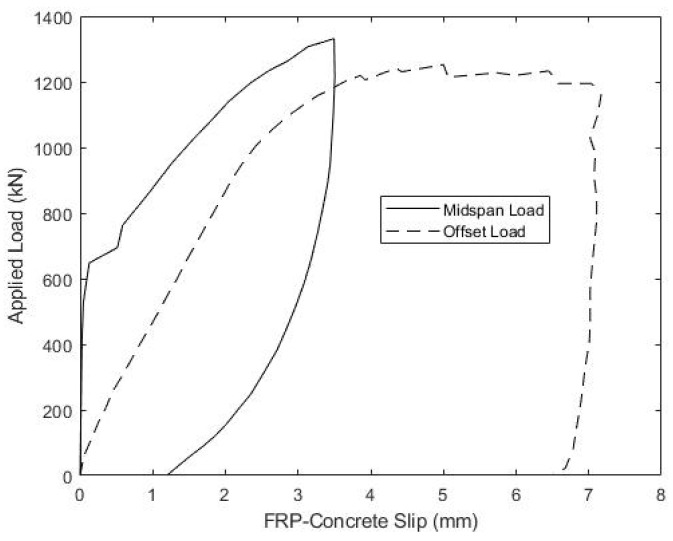
Beam B2 Ultimate Load-Slip Response.

The relationship between the applied load and slip between the concrete deck and girder top flange is also of significant interest. Figure 16 and Figure 17 show the measured load versus the average slip for both the midspan and offset load scenarios for beams B1 and B2, respectively. Both specimens showed consistently small slips and relatively linear responses, until the midspan loads of about 650 kN, which corresponds to a shear of 325 kN and an interface shear flow, $f_{v}^{u}$, of 0.355 kN/mm. At larger loads, slips increased more quickly to a maximum average value of 3–3.5 mm. During subsequent offset loading, both B1 and B2 exhibited nearly linear load-slip responses until about 950 kN of the applied load, which corresponded to a slip of ~2 mm. The softer interface response, measured during the offset loading, does indicate that loads larger than 650 kN in the midspan load test might have caused some damage to the girder-deck interface.

While girder-deck interface slips measured during the offset loading test are larger than those recorded during the compression-shear tests, they cannot be directly compared, due to the different specimen scales and lengths of the deck-flange interface in the two tests. It is also important to emphasize that the AASHTO LRFD Strength I factored interface shear is 0.404 kN/mm, which is only 14% above the value of $f_{v}^{u}$ = 0.355 kN/mm, corresponding to the apparent onset of damage, and is less than half of the average $f_{v}^{u}$ at beam failure.

## 7. Assessment of Stainless Steel Connectors

### 7.1. Specimen Details and Loading Protocol

While the shear block and beam tests detailed previously demonstrated good connector performance, several practical improvements are worthy of exploration. First, the use of a stainless steel threaded rod, in lieu of structural bolts, would significantly increase the connector corrosion resistance. While stainless steel typically possesses reduced fatigue resistance, which will be magnified by the inclusion of threads in the shear plane, its use might be feasible, given that girder-deck shear flow is transferred through mechanical interlock between the girder flange and deck, rather than through the connectors beating on the holes in the top flange. Second, the connector pre-tension used in the previous specimens will result in significant additional labor in field applications that might not be necessary, and there is no guarantee that connector pre-tension will be retained over the life of a structure. Third, the self-consolidating concrete mix, used in all the previous tests, is not typical for a conventional cast-in-place concrete deck.

To address these issues, two additional shear block specimens (S5 and S6) were fabricated and fatigued for 6 × 10^6^ load cycles, prior to being loaded to failure. The specimens and loading protocol were identical to the previously detailed compression-shear tests, except for key differences described next and summarized in Table 4. First, both specimens S5 and S6 were fabricated with a 25.4 mm diameter, 18-8 (grade 304) stainless steel rods with a nominal yield stress of 215 MPa and a nominal ultimate tensile strength of 505 MPa. The rods were not pre-tensioned to the level of the previous tests, and the nuts were only lightly torqued. Second, a normal concrete mix with a 20 mm maximum aggregate size, typical for cast-in-place concrete decks, was used for both specimens. The concrete compressive strength of both specimens on the day of strength testing was 74.5 MPa, as reported in Table 4, but ranged from 35.6 MPa (specimen S6) to 45.9 MPa (specimen S5) at the start of fatigue testing. Third, to accommodate the larger aggregate, the FRP plate ridge amplitude was increased to 12.7 mm. Finally, slip was not recorded during the post-fatigue tests to failure.

### 7.2. Stainless Steel Connector Test Results

**Figure 18:**
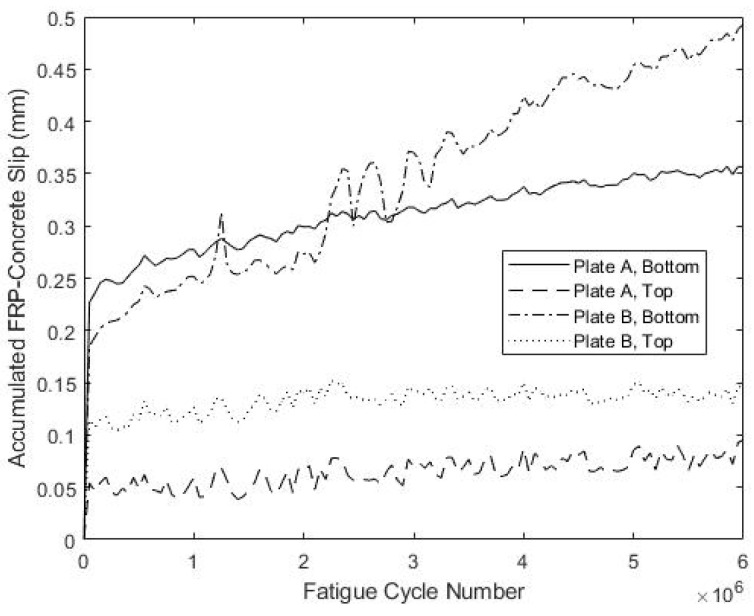
Fatigue-Induced Slip at FRP-Concrete Interface for S5.

**Figure 19:**
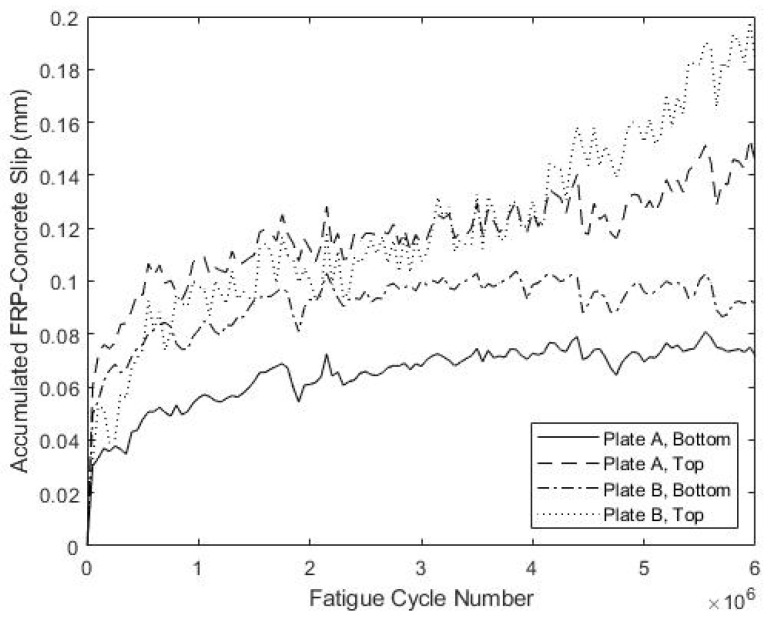
Fatigue-Induced Slip at FRP-Concrete Interface for S6.

The development of the connector slips during fatigue testing is shown in Figure 18 and Figure 19 for specimens S5 and S6, respectively. For S5, three of the four measured slip values stabilized after about 1 × 10^6^ cycles and increased little thereafter, whereas for S6, this was the case for two out of four measured slip values. The maximum measured slip at the bottom of plate B for specimen S5 was 0.49 mm, which is larger than the maximum observed slip for specimens S1–S4. However, the large oscillations in measured slip at this location over 2.5 × 10^6^–3.5 × 10^6^ cycles might reflect poor anchoring of the LVDT at that location. Specimen S6 exhibited smaller slips that never exceeded 0.2 mm, which is more consistent with the results from specimens S1–S4. Overall, these results indicate performance during load cycling similar to that observed for specimens S1–S4.

**Figure 20:**
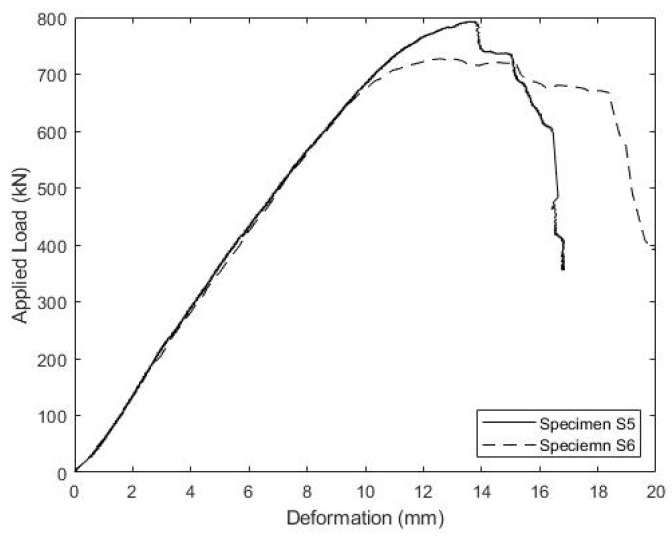
Stainless Steel Connector Load-Deformation Response under Monotonic Loading to Failure.

Figure 20 includes the load vs load head displacement relationship during the post-fatigue failure tests. Specimen S5 had an ultimate strength of 791 kN, while S6 failed at 729 kN, although S6 exhibited more ductility with a longer load plateau. As with previous specimens, the peak load was characterized by the shear failure of the concrete ridges, and the lower concrete compressive strength of specimen S6 could explain its somewhat lower capacity and greater ductility. The shear flows at failure are given in Table 4, and are less than the shear flows at failure observed for specimens S1–S3, but greater than those for S4. However, specimens S1–S3 all had higher concrete strengths than specimens S5 and S6, whereas specimens S5 and S6 both had higher concrete strengths than specimen S4. The normalized capacity, $f_{v}^{u}/\sqrt{f_{c}^{\prime }}$, given in the last column of Table 4 indicates values somewhat lower than those observed for specimens S1–S4. However, this could be due to the fact that, unlike specimens S5 and S6, the connectors in specimens S1–S4 were consistently pre-tensioned to a high level. This pre-tension will induce compressive stress on the shear plane, tending to increase the concrete strength. Relative to the AASHTO LRFD Strength I factored demand for the model bridge girder of 0.404 kN/mm, specimen S5 exhibited 75% excess capacity and specimen S6 exhibited 61% excess capacity. Furthermore, given a yield stress of 215 MPa for the stainless steel rod, Equation (2) predicts a capacity of 0.321 kN/mm for both specimens, assuming a connector spacing of 30.5 cm, which is less than half the measured capacity of both specimens. These low predicted strengths are due to the low yield stress of the 304 stainless steel, and the discrepancy with the observed values could indicate that the connector yield strength does not play a significant role in strength, as implied by Equation (2). This is consistent with the post-mortem observations of concrete ridge shear failure. The connection stiffness (load vs. slip) cannot be determined, since slips were not measured during the strength tests.

## 8. Summary and Conclusions

This paper has detailed the development of a new, friction-type connector designed to transfer shear between the top flange of an FRP tub girder and concrete deck used in bridge applications. The connection’s configuration and load transfer mechanism are based on the current code provisions for the design of conventional shear-friction connections that are widely used in concrete structures. Four compression-shear specimens were tested to assess the connector fatigue resistance and ultimate strength. Compared to previously used bearing-type connectors, this system is materially efficient, simple to fabricate with no reliance on secondary bonding or welding, provides a high degree of interface stiffness, and can be fabricated with low-grade structural or stainless steel fasteners. Based on the compression-shear tests and short beam tests, the connection exhibited strength exceeding that predicted by AASHTO for frictional concrete-concrete connections. The connection strengths were also between 54% and 122% greater than the factored demand required by AASHTO for a typical model bridge girder. The cyclic loading of the connection in both compression-shear and bending showed that connection stiffness and strength do not significantly degrade, due to the application of 1 × 10^6^ to 6 × 10^6^ cycles of factored fatigue load.

The consistent failure of the specimens by concrete ridge shearing suggested that concrete shear strength governs the connection strength as a whole, with FRP ridges providing adequate interlock to connection failure. This is supported by the tight range of failure loads within each specimen type after normalization by concrete shear strength. The comparison of normalized failure loads across specimen types indicates that the presence of net compression on the plane of the connection might also enhance capacity. However, the number of tests is insufficient to draw definitive conclusions regarding the impact of concrete strength and net compression on capacity.

Despite the good performance of the connector that indicates suitability for bridge construction, unanswered questions and potential for improvement remain, as summarized below.

1. The results indicate that concrete strength has a significant impact on connection capacity, although the number and variety of tests performed here is not sufficient to develop a relationship between concrete strength and connection capacity. Additional testing that captures a range of typical concrete strengths would be valuable.
2. While the connectors exhibited both pre- and post-fatigue strength in excess of that predicted by AASHTO provisions, connection capacity was governed by concrete ridge shear failure, which is not explicitly addressed in the AASHTO provisions. Given this observation and the mechanical interlocking nature of the connection, it is possible that alternative fasteners, such as lower-grade stainless steel or even FRP bars, could give adequate performance. This should be assessed in future studies.

## 9. Patents

The first author is a co-inventor of the FRP bridge girder covered by the U.S. Patent No. 10,494,779, which includes some details of the shear connection described and assessed here.

## Figures and Tables

**Table 1:** FRP Mechanical Design Properties.

Component	Infused Thickness (mm)	Longitudinal Modulus *E* (GPa)	Ultimate Compressive Stress *F_c_* (MPa)	Ultimate Shear Stress *τ* (MPa)	Ultimate Tensile Stress *F_t_* (MPa)
Top Flange	25.4	27.1	375	NA	NA
Web	4.9	15.4	NA	68.9	205
Bottom Flange	18.8	77.5	NA	NA	765

**Table 2:** Summary of A325 Connector Specimen Test Protocols and Strength Test Results.

Specimen Number	$f_{c}^{\prime }\;(\mathrm{MPa})$	Fatigued	$P_{min}\;(\mathrm{kN})$	$P_{max}\;(\mathrm{kN})$	Failure Load (kN)	$\mathrm{Shear}\;\mathrm{Flow}\;\mathrm{at}\;\mathrm{Failure}\;f_{v}^{u}\;(\mathrm{kN}/\mathrm{mm})$	$\frac{f_{v}^{u}}{\sqrt{f_{c}^{\prime }}}$
S1	84.8	Yes	44.8	212	927	0.829	90.0
S2	84.8	Yes	44.8	212	915	0.818	88.8
S3	84.8	No	NA	NA	872	0.780	84.7
S4	51.1	No	NA	NA	698	0.624	87.3

**Table 3:** Key Dimensions of Short Beam Shear Specimens.

Overall Dimensions	Depth	27.9 cm
	Out-to-Out Flange Width	65.4 cm
	Length	2.44 m
Top Flange	Thickness	2.54 cm
	Width	15.2 cm
Web	Depth	22.9 cm
	Thickness (excluding foam core)	2.54 cm
	Angle (from vertical)	9.9 deg
Bottom Flange	Thickness	2.54 cm
	Width	16.5 cm

**Table 4:** Summary of Stainless Steel Connector Specimen Test Protocols and Strength Test Results.

Specimen Number	$f_{c}^{\prime }\;(\mathrm{MPa})$	Fatigued	$P_{min}\;(\mathrm{kN})$	$P_{max}\;(\mathrm{kN})$	Failure Load (kN)	$\mathrm{Shear}\;\mathrm{Flow}\;\mathrm{at}\;\mathrm{Failure}\;f_{v}^{u}\;(\mathrm{kN}/\mathrm{mm})$	$\frac{f_{v}^{u}}{\sqrt{f_{c}^{\prime }}}$
S5	74.5	Yes	129	316	792	0.708	82.0
S6	74.5	Yes	129	316	729	0.652	75.5

## Data Availability

Data reported herein are available by request to the corresponding author, W.D.
